# Frequent emergency department users in Finnish public healthcare – a nationwide registry-based study

**DOI:** 10.1186/s12913-026-14049-5

**Published:** 2026-01-20

**Authors:** Anna-Maaria Vähä, Marja-Leena Lamidi, Mika Linna, Katja Wikström, Tiina Laatikainen

**Affiliations:** 1 University of Eastern Finland, P.O. Box 1627, Kuopio, FI-70211 Finland; 2 Wellbeing Services County of North Karelia Finland – Siun sote, Tikkamäentie 16, Joensuu, FI-80210 Finland; 3 Finnish Institute for Health and Welfare, P.O. Box 30, Helsinki, FI-00271 Finland

**Keywords:** Frequent emergency department use, Multimorbidity, Chronic diseases, Health service utilisation

## Abstract

**Background:**

Frequent users of emergency departments (ED) often have complex healthcare needs and chronic conditions, contributing markedly to the overall ED burden. This study aimed to characterise frequent ED users in Finnish public healthcare and study frequent ED use among patients with chronic diseases.

**Methods:**

This was a nationwide registry-based cross-sectional analysis using national register data from the Finnish Care Register and Statistics Finland, we analysed all adults (≥ 18 years) who used public healthcare services in 2018 (*N* = 3.05 million). ED users were classified as frequent (≥ 4 visits/year) or infrequent. Logistic regression assessed associations between frequent ED use and variables including age, sex, region, multimorbidity, and prior healthcare use. Spearman correlation evaluated associations with 2017 service use.

**Results:**

In 2018, 1.4% (41,880 individuals) were frequent users, contributing 24.3% of all ED visits. Frequent users were older (mean age 65 vs. 55 years) and more often multimorbid (30.5% vs. 10.1%) than infrequent users. Prior ED use was the strongest predictor of future frequent use (Odds Ratio (OR) 22.43). Multimorbidity increased the odds of frequent ED use (OR 4.26; 95% CI: 4.15–4.38, *p* < 0.001). Psychiatric and behavioural disorders related to substance abuse (14.4%), chronic kidney disease (6.5%) were among the conditions with the most frequent ED use. The most common patients among all ED users were those with hypertensive diseases (21.9%), other diseases of the heart and pulmonary circulation (10.3%), and they also had a high prevalence of frequent ED use 2.9% and 5.6% respectively.

**Conclusions:**

A subgroup analysis of frequent ED users, particularly those with multimorbidity and chronic cardiac, pulmonary, or psychiatric conditions, can inform policies for integrated care and resource allocation. Targeted interventions and improved coordination across primary, specialised, and social care could reduce ED use and overall system burden.

**Supplementary Information:**

The online version contains supplementary material available at 10.1186/s12913-026-14049-5.

## Introduction

The use of ED services has increased markedly in recent decades in many high-income countries [1], driven by population ageing, the rising prevalence of chronic diseases, and growing pressures on health and social care systems [2]. A small but heterogeneous subset of patients use ED services repeatedly; although they represent only 4.5–8% of ll ED users, they account for 21–30% of all visits [3]. Frequent ED users typically have complex health needs, multimorbidity [4, 5], and high utilisation of other healthcare services [3, 4, 6]. Multimorbidity is particularly relevant in ageing societies, including the Nordic countries, where chronic cardiometabolic, respiratory, renal, and mental health conditions are major contributors to service demand [2, 5]. Increased visits to primary and specialised care, emergency departments, and hospital admissions among patients with multimorbidity have all been linked to rising healthcare costs [7].

Evidence suggests that limitations in primary care accessibility, continuity, and care coordination can contribute to ED attendance among patients with multimorbidity or complex health and social needs [7–9]. Inappropriate or non-urgent ED use can exacerbate overcrowding, prolong waiting times, and increase healthcare costs [10]. Such visits are more common among frequent ED users than among infrequent ED (less than four visits/year) users [9]. Parkinson et al. [11] further classified avoidable ED attendances as divertible, preventable, or unnecessary from a system perspective. Despite frequent ED users being a well-studied group, most research has been conducted with restricted and small patient populations. Therefore, more studies using representative data from large, unselected patient groups are needed.

Although frequent ED use has been widely recognised as an international challenge, most previous research has been limited to single hospitals, specific patient groups, or regional samples. Consequently, there remains a need for studies using large, unselected, population-level data to better understand patterns of frequent ED utilisation. Managing multimorbidity is difficult for patients and health systems alike, and current models of care, traditionally organised around single diseases, are often poorly suited to meet these needs [12]. Healthcare systems that can effectively respond to the growing burden of multimorbidity are required, alongside country-specific evidence on its prevalence and impact on service use.

This study aimed to identify the characteristics of frequent ED users in Finnish public healthcare and to examine how chronic diseases, multimorbidity, and prior healthcare utilisation are associated with frequent ED attendance in a nationwide sample. We hypothesised that frequent ED use would be more common among individuals with multimorbidity, older people and those who had higher prior healthcare use.

## Methods

The data for this study was obtained from the Finnish Care Register for Healthcare for the years 2015–2018 [13] and from Statistics Finland. These national registers include information on the patients’ age, sex, and all recorded contacts with public healthcare services. Each encounter with a healthcare professional is documented as a separate entry, including the type of contact and the related diagnoses. We extracted data on age, sex, use of public healthcare services, and diagnoses coded according to the International Classification of Diseases, 10th Revision (ICD-10).

The study population comprised all adults aged 18 years or older who lived in Finland and used public primary or specialised healthcare services in 2018 (excluding the Åland Islands). A total of 3.05 million individuals were included. Participants were categorised by sex (women and men) and into four age groups: 18–24, 25–64, 65–79, and ≥ 80 years. Data from 2015 to 2017 was used to determine the baseline morbidity status. The classification of major chronic disease groups was based on a previously published multimorbidity study [14]. Supplementary Table 1 lists the disease groups and corresponding ICD-10 codes.

Morbidity was defined using all diagnoses recorded in public primary and specialised care. Individuals with diagnoses belonging to two or more chronic disease groups were classified as multimorbid. Previous healthcare use was assessed for all patients and included as a covariate to evaluate its association with frequent ED use. Healthcare use was described using eight non-mutually exclusive categories: frequent ED use; hospitalisation in a specialised care ward; one outpatient visit to specialised care; two or more outpatient visits to specialised care; hospitalisation in a primary care ward; one outpatient visit to primary care; two to five outpatient visits to primary care; and six or more outpatient visits to primary care. Each patient received values for all applicable service-use categories.

Finland has a universal public healthcare system organised into 21 wellbeing services counties, which since 2023 have been responsible for all health, social, and rescue services previously organised by municipalities and hospital districts [15]. These counties are primarily funded by the central government [15]. Public hospitals provide nearly all ED services in Finland [16]. Private hospitals offer elective specialist care, diagnostics, and outpatient services, but emergency care for life-threatening conditions is the responsibility of the public system [17].

Client fees in public healthcare vary slightly by wellbeing services county, but national maximum rates apply. A primary care physician visit costs up to 28.20 euros (EUR), and an outpatient specialist or hospital clinic visit approximately 66.70 EUR. An ED visit fee may be charged from individuals aged 18 years or older, with a maximum of 38.70 EUR per visit. Hospital inpatient care carries a daily fee of 66.90 EUR, and annual payment caps (approximately 762 EUR in 2024) limit out-of-pocket expenditures [18].

ED visits were identified from the register using service sector code 91, which denotes emergency care in specialised healthcare. This code captures all visits in Finland’s 24/7 joint emergency duty model, where primary and specialised emergency services are integrated and provided mainly in hospital-based EDs. Because each healthcare contact is registered as a separate entry, all encounters meeting these criteria were included without additional exclusions.

Frequent ED use was defined as four or more ED visits within 12 months, a widely used definition in the international literature [9, 19–22].

### Statistical analysis

The proportions of patients within each chronic disease group (2015–2017) among all ED users, as well as the proportions of frequent ED users within each ICD-10-based chronic disease category, were calculated. Differences between frequent and infrequent ED users in counts, percentages, and age were examined using the Mann-Whitney U test, chi-squared test, and independent samples t-test, as appropriate. Logistic regression was applied to assess the association between frequent ED use and potential explanatory variables (sex, age group, sex*age group interaction, wellbeing services county, and previous healthcare use), all entered as fixed factors. Spearman correlation, a robust method for skewed distributions, was used to estimate the magnitude of unadjusted associations between the ED visit frequency and other healthcare use. Statistical analyses were performed using R (version 4.1.3) [23]. A p-value < 0.05 was considered statistically significant, and 95% confidence intervals (CI) were reported for all odds ratios.

## Results

In 2018, 606,950 individuals (19.9% of all public healthcare users) had at least one ED visit in Finland. Of all healthcare users, 1.4% were frequent ED users (≥ 4 visits/year), representing 6.9% of all ED users. These individuals accounted for 234,697 visits, corresponding to 24.3% of all ED contacts (Table 1). Infrequent ED users were younger (mean age 55 years) than frequent users (mean age 65 years), and women were slightly more represented among frequent users.

Among frequent ED users, 30.5% were multimorbid, and an additional 27.2% had one chronic disease group diagnosis. Multimorbid patients accounted for 20.5% of all ED visits. Compared with individuals without chronic disease, multimorbidity was strongly associated with frequent ED use (OR 4.26; 95% CI 4.15–4.38, *p* < 0.001), and having one chronic condition also increased the likelihood of frequent ED attendance (OR 2.14; 95% CI 2.09–2.19).

**Table 1:** Characteristics of frequent and infrequent emergency department (ED) users in 2018

	Frequent ED users	Infrequent ED users
Visits to ED in 2018 (n)	234,697	767,365
Patients with ED visit (n)	41,880	565,070
ED visits per patient (n)	5.5	1.3
Proportion of women (%)	56.5	54.8
Proportion of men (%)	43.5	45.2
Age (mean, years)	65	55
Patients with multimorbidity (%)	30.5	10.1
Patients with one chronic disease (%)	27.2	19.1
Patients with no chronic diseases (%)	42.3	70.8

Values are presented as numbers (n), percentages (%), or means as appropriate. Group differences from row 3 downward were all statistically significant (p < 0.001). Mann–Whitney U-test was used for ED visits per patient, χ² test for proportions, and independent samples t-test for age

Across diagnostic groups based on healthcare use from 2015 to 2017, the highest proportions of frequent ED users in 2018 were observed among patients with psychiatric and behavioural disorders related to substance abuse (14.4%), chronic kidney and urinary tract diseases (6.5%), schizophrenia and delusional disorders (6.4%), cerebrovascular diseases (6.3%), and other cardiac and pulmonary circulation diseases (5.3%) (Table 2). The full table including all diagnostic categories and percentages is available in Supplementary Table 2.

In contrast, the largest diagnostic groups among all ED users were hypertensive diseases (21.9%), other diseases of the heart and pulmonary circulation (10.3%), diabetes (4.0%), diseases of male genitals/reproductive organs (3.5%), and chronic lower respiratory diseases (2.8%). Only 0.1% of all ED users had diagnoses of psychiatric and behavioural diseases related to substance abuse despite their high prevalence.

**Table 2:** Proportion of frequent ED users within the ten most common specific patient groups and the proportion of these patients among all ED users in 2018

Disease group*	The proportion of frequent ED users within a specific patient group (%)	The proportion of these patients among all ED service users (%)
Psychiatric and behavioural diseases related to substance abuse	14.4	0.1
Chronic diseases of the kidneys and urinary tract	6.5	0.7
Schizophrenia and delusional diseases	6.4	1.0
Cerebrovascular diseases	6.3	0.3
Other diseases of the heart and pulmonary circulation	5.3	10.3
Blood diseases and blood-forming organs	4.7	0.3
Dementia and organic psychical disorders	4.6	1.2
Ischemic heart diseases	4.5	1.1
Epilepsy and migraine	4.2	0.5
Diseases of nerves and nerve-muscle junction	4.2	0.1

* Definitions of the disease groups appear in Supplementary Table 1. Complete results for all chronic disease categories are provided in Supplementary Table 2

In the logistic regression models presented in Table 3, all explanatory variables were statistically significant (*p* < 0.001), although they displayed modest predictive power (Nagelkerke R^2 varying from 0.054 to 0.103 for models with one explanatory variable and 0.111 for the multivariate model). The prevalence of frequent ED use increased markedly with age, reaching 3.2% among those ≥ 80 years compared with those aged 65–79 years (1.5%). Women had the highest relative share of frequent users in the youngest 18–24 years (1.3%) and oldest ≥ 80 years (3.0%) groups, while men had higher proportions in the 65–79 and ≥ 80 age groups (1.7% and 3.4%, respectively). Overall, frequent ED use was slightly more common in men (1.4%) than in women (1.3%).

The number of ED visits in 2018 correlated most strongly with hospitalisations in specialised care in 2017 (Spearman *r* = 0.533). Weaker correlations were observed for primary care ward stays (*r* = 0.272), other specialised care use (*r* = 0.238), and primary care outpatient visits (*r* = 0.162). For frequent ED use, the strongest association was with prior ED visits (*r* = 0.211). All correlations were statistically significant (*p* < 0.001) due to the large sample size.

These zero-order correlations represent unadjusted associations and naturally reflect the fact that service use tends to cluster within individuals and is also related to age and sex. When controlling for these factors and for other service-use variables in the logistic regression, several effect estimates decreased from Model 1 to Model 2 (Table 3), as expected when covariates share variance in the same direction.

Consistent with the correlation results, the logistic regression demonstrated strong associations between prior healthcare utilisation and frequent ED use in 2018 (Table 3). Notably, having ≥ 4 ED visits in 2017 was the strongest predictor of frequent ED use in 2018 (Model 1 OR 22.02; 95% CI 21.42–22.66). Additional healthcare utilisation (hospitalisations or frequent primary or specialised outpatient visits) showed graded associations with frequent ED attendance.

**Table 3:** Association between healthcare use in 2017 and frequent ED use in 2018: logistic regression models

Use of healthcare services in 2017	Model 1 OR (95% CI)	*R*^2	Model 2 OR (95% CI)
**Hospitalisation in the special healthcare ward**: yes vs. no	5.46 (5.35–5.57)	0.090	2.88 (2.81–2.95)
**Hospitalisation in the primary healthcare ward**: yes vs. no	5.08 (4.93–5.23)	0.054	1.68 (1.63–1.74)
**Number of outpatient visits to special healthcare**		0.069	
Outpatient visits to specialised care: 1 vs. 0	1.87 (1.81–1.94)		1.44 (1.39–1.49)
Outpatient visits to specialised care: ≥2 vs. 0	3.84 (3.76–3.93)		2.19 (2.14–2.25)
**Number of outpatient visits in primary care**		0.060	
Outpatient visits to primary care: 1 vs. 0	1.13 (1.08–1.18)		1.05 (1.00–1.09)
Outpatient visits to primary care: 2–5 vs. 0	1.61 (1.55–1.66)		1.25 (1.20–1.29)
Outpatient visits to primary care: ≥6 vs. 0	3.91 (3.79–4.04)		1.97 (1.90–2.04)
Frequent ED use in 2017 (≥ 4 vs. 0–3)	22.02 (21.42–22.66)	0.103	

Model 1: Each service-use variable tested separately, adjusted for sex, age group, sex×age interaction, and wellbeing services county. Nagelkerke R^2 shortened as R^2

Model 2: All service-use variables included simultaneously with the same adjustments. Nagelkerke R^2 0.111 for model 2.

Nagelkerke R² values represent model fit. OR = odds ratio; CI = confidence interval

## Discussion

This study examined the characteristics of frequent ED users in Finnish public healthcare and the associations between chronic diseases, multimorbidity and prior service use with repeated ED attendance. The findings support multimorbidity as an important risk factor for frequent ED use. Consistent with earlier research, chronic conditions were more common among frequent ED users than among the general population [24], and multimorbid patients had a substantially higher utilisation of health services [25]. Multimorbid patients are typically older, have higher health service use as well as increased risks of hospitalisation and mortality compared to healthy populations or patients with only one chronic condition [25].

Frequent ED use was most common in older age groups, mirroring earlier studies [22, 26, 27]. Recent Finnish evidence from the Vitality 90 + Study by Abraham et al. [28] showed that ED use remains common among the oldest old and is driven by multimorbidity, functional limitations and high prior service use. Prior research has shown that older frequent ED users often have multimorbidity [21, 26, 28], chronic kidney disease [27] and cardiac conditions [29]. In our study, chronic cardiac and pulmonary diseases were among the most common diagnoses among ED users, and these groups had one of the highest proportions of frequent ED use. Similar patterns have been observed in ageing European populations [5, 30]. Evidence suggests that integrated, continuous care can reduce avoidable ED visits among older adults [31, 32], while Finnish findings indicate that proactive primary care screening reduces ED use and related costs among home-dwelling older adults [31].

Mental health [32] and substance use related conditions were also strongly linked to frequent ED attendance, as shown previously [22]. Alcohol and substance use disorders in particular are known to drive heavy ED utilisation [22, 33]. Our findings support this: although patients with substance use related psychiatric diagnoses accounted for a small share of all users, they had by far the highest prevalence of frequent ED use. This aligns with national evidence showing that individuals with alcohol problems often bypass planned services and rely on EDs [33], as well as qualitative research describing unmet needs and poor continuity of care among patients with severe mental disorders [34].

These findings also correspond with subgroup analyses in earlier studies, such as Dufour et al., who identified frequent ED user profiles ranging from low-comorbidity patients to those with cardiac, pulmonary, dementia or psychiatric conditions [29]. Some frequent ED users with low comorbidity may reflect social rather than medical needs [26]. A Swedish study similarly found that older frequent ED users often experienced social disadvantages [21]. Future research should explore social-care and integrated service models for these groups.

Strong associations between prior healthcare use and later ED use were observed, consistent with prior work showing the predictive value of previous hospitalisations and primary care utilisation [4, 21, 22, 35]. The association between ≥ 4 ED visits in 2017 and frequent ED use in 2018 remained high, consistent with earlier US findings [19]. As expected, crude Spearman correlations were larger than adjusted model effects because healthcare utilisation correlates with age, sex, and other service-use categories. When these covariates were included in Model 2, the effect sizes decreased, reflecting shared variance between explanatory factors rather than reduced substantive importance.

Finland is among the most rapidly ageing populations in Europe [36], and the prevalence of chronic conditions continues to grow. At the same time, insufficient capacity in primary care [37, 38] and mental health/substance use services [39] may lead patients to rely on EDs when timely outpatient care is unavailable. Fragmented care pathways and limited coordination between primary care, specialised care and social services can further reinforce ED dependence [8, 27, 34].

## Strengths and limitations

A major strength of this study is the use of large, nationwide longitudinal data sets covering both public primary and specialised healthcare, enabling a broad and highly representative analysis of service utilisation, particularly ED use. Although the diagnostic validity of the Finnish Care Register for Healthcare is generally high [13], some minor misclassification cannot be entirely ruled out. However, given the well-documented completeness and accuracy of the Finnish Care Register for Healthcare and the large, unselected study population, any such misclassification is unlikely to meaningfully affect the findings. Including chronic disease groups further strengthens the study by highlighting how complex health conditions shape frequent ED attendance and helping identify subgroups with high service needs and complex care pathways.

This study also has limitations. Numerous socioeconomic and sociodemographic factors have been linked to frequent ED use, but the Finnish Care Register for Healthcare contains no variables beyond age and sex. Broader indicators, such as income, education or other socioeconomic or socio-demographic were not available and therefore could not be included. Although private healthcare contacts are not fully captured in the national registers, this does not meaningfully affect our findings because 24/7 emergency department services in Finland are provided exclusively by the public sector [16, 17]. The cross-sectional design limits causal inference. The relatively low Nagelkerke’s R² values suggest that, although the included factors are statistically associated with frequent ED use, they explain only a small portion of the variance. This highlights the complexity of healthcare utilisation and the likely impact of unmeasured clinical, social, and system-level determinants. In addition, there is no universally accepted definition of frequent ED use; thresholds in previous research vary from three to twelve annual visits, which may complicate comparisons and limit generalisability. The data also do not distinguish between avoidable and clinically necessary ED visits, restricting the interpretation of appropriateness. Finally, the lack of cost information prevents analysis of the economic burden associated with frequent ED use.

## Conclusion

Based on this study of 3.05 million individuals, frequent ED users constitute a heterogeneous group with diverse needs and drivers of repeat visits. Although they represented only 1.4% of all healthcare users, they accounted for nearly one quarter of all ED visits, underscoring the disproportionate burden posed by this small subgroup and the potential impact of targeted interventions. Strengthening continuity in primary care, improving access to mental health and substance use services, and developing coordinated, multidisciplinary care models for people with multimorbidity may help reduce preventable ED use. Early identification of patients with repeated ED visits, followed by proactive and structured follow-up, could support more integrated care pathways and ease pressures on emergency services. Our findings indicate that focused analyses of key subgroups such as long-term frequent ED users, individuals with multimorbidity, and those with cardiac or pulmonary diseases may help identify avoidable visits and guide cost-effective service development. 

## Supplementary Information

Below is the link to the electronic supplementary material.

Supplementary Material 1

Supplementary Material 2

## Data Availability

The data used in this study comes from the registers of the Finnish Institute for Health and Welfare. These data sets are not publicly available, because their use is restricted by Finnish data protection legislation. The data was accessed under a license granted specifically for this study. According to Finnish legislation, register authorities may grant permission to use register data, including sensitive personal information such as health data, only for defined research questions. Permission is given to named researchers who must sign a pledge of secrecy, and the data cannot be shared with others. Other researchers can apply for access to these data sets through the Health and Social Data Permit Authority Findata, which processes data permit applications concerning the registers of the Finnish Institute for Health and Welfare. https://www.findata.fi/en/services/data-requests/.

## **Abbreviations**

CI: Confidence interval
ED: Emergency department
EUR: Euro
ICD-10: International Classification of Diseases, 10th Revision
OR: Odds Ratio
