# Supplementary material for: Frequent emergency department users in Finnish public healthcare – a nationwide registry-based study
Source: BMC Health Serv Res. 2026 Jan 20;26:246. doi: 10.1186/s12913-026-14049-5 (PMC12905971; doi:10.1186/s12913-026-14049-5)
Supplement: Supplementary file 1 — Supplementary Material 1 [file 12913_2026_14049_MOESM1_ESM.docx]

**Supplementary Table 1. Disease groups and included diagnosis (ICD-10).**

| Disease group | ICD-10 codes |
| --- | --- |
| 1) Cancer and in-situ carcinomas | C00—C97, D00—D09 |
| 2) Blood diseases and blood-forming organs | D50—D53, D55—D61, D63, D64, D66—D69, D71—89 |
| 3) Endocrine diseases | E00—E03, E05—E07, E20—E29, E31, E34, E35 |
| 4) Diabetes | E10—E14 |
| 5) Obesity and other metabolic diseases | E65, E66, E68, E70—E72, E74—E80, E83—E85, E88—E90 |
| 6) Dementia and organic psychical disorders | F00—F09, G30—G32 |
| 7) Psychiatric and behavioural diseases related to substance abuse | F10—F16, F18, F19 |
| 8) Schizophrenia and delusional diseases | F20—F22, F24, F25, F28, F29 |
| 9) Mood disorders | F30—F39 |
| 10) Neurotic, stress-related and somatoform diseases, including eating disorders | F40—42, F44, F45, F48, F50, F52— F55, F59—F63, F68, F69 |
| 11) Sleep disorders | F51, G47 |
| 12) Other neurological diseases | G10—G13, G20—G26, G35, G37 |
| 13) Epilepsy and migraine | G40—G44 |
| 14) Diseases of nerves and nerve-muscle junction | G50—G60, G62—G64, G70—G73 |
| 15) Chronic eye diseases and blindness | H17—H21, H25—H28, H31, H33, H35, H36, H40—H42, H47—H49, H51, H54 |
| 16) Chronic ear diseases and deafness | H80—H83, H90, H91, H93, H95 |
| 17) Hypertensive diseases | I10—I15, I95—I99 |
| 18) Ischemic heart diseases | I20—I25 |
| 19) Other diseases of the heart and pulmonary circulation | I27, I28, 131, I34—I39, I42—I45, I48—I51 |
| 20) Cerebrovascular diseases | I60—I69 |
| 21) Diseases or arteries and veins | I70—I73, I78, I79, I83—I87, I89 |
| 22) Chronic diseases of the upper respiratory tract | J30—J35, J37, J38 |
| 23) Chronic diseases of the lower respiratory tract | J41—J47, J60—J68, J70, J84, J92, J95—J99 |
| 24) Diseases of the oesophagus, stomach, and duodenum | K21—K31 |
| 25) Inflammatory and other bowel diseases | K50—K52, K55, K57—K59, K62, K63, K90 |
| 26) Diseases of the liver, pancreas, and biliary tract | K70—K77, K80, K81, K86 |
| 27) Chronic skin diseases | L10, L12, L13, L20, L21, L23, L24, L26, L28, L40, L41, L43—L45 |
| 28) Inflammatory diseases of joints and connective tissue | M05—M14, M30—M36 |
| 29) Arthrosis | M15—M19 |
| 30) Other musculoskeletal diseases | M20—M24, M70, M72—M75, M79—M84, M91, M93, M94, M96, M99 |
| 31) Back diseases | M40—M54 |
| 32) Chronic diseases of the kidneys and urinary tract | N01, N03—N05, N07, N08, N11, N18—N22, N31-N33, N35 |
| 33) Diseases of male genitals/reproductive organs | N40—N42, N48, N50 |
| 34) Diseases of female genitals/reproductive organs | N80, N81, N88 |
