# Supplementary material for: Frequent emergency department users in Finnish public healthcare – a nationwide registry-based study
Source: BMC Health Serv Res. 2026 Jan 20;26:246. doi: 10.1186/s12913-026-14049-5 (PMC12905971; doi:10.1186/s12913-026-14049-5)
Supplement: Supplementary file 2 — Supplementary Material 2 [file 12913_2026_14049_MOESM2_ESM.docx]

**Supplementary table 2. The proportion of frequent ED users within a specific patient group and the proportion of these patients among all ED service users in 2018**

| Disease group* | The proportion of frequent ED users within a specific patient group (%) | The proportion of these patients among all ED service users (%) |
| --- | --- | --- |
| Psychiatric and behavioural diseases related to substance abuse | 14.4 | 0.1 |
| Chronic diseases of the kidneys and urinary tract | 6.5 | 0.7 |
| Schizophrenia and delusional diseases | 6.4 | 1.0 |
| Cerebrovascular diseases | 6.3 | 0.3 |
| Other diseases of the heart and pulmonary circulation | 5.3 | 10.3 |
| Blood diseases and blood-forming organs | 4.7 | 0.3 |
| Dementia and organic psychical disorders | 4.6 | 1.2 |
| Ischemic heart diseases | 4.5 | 1.1 |
| Epilepsy and migraine | 4.2 | 0.5 |
| Diseases of nerves and nerve-muscle junction | 4.2 | 0.1 |
| Inflammatory diseases of joints and connective tissue | 4.1 | 0.7 |
| Chronic diseases of the lower respiratory tract | 4.0 | 2.8 |
| Mood disorders | 4.0 | 1.5 |
| Diseases of the liver, pancreas, and biliary tract | 4.0 | 0.1 |
| Neurotic, stress-related and somatoform diseases, incl. eating disorders | 3.9 | 0.4 |
| Other neurological diseases | 3.8 | 1.3 |
| Cancer and in-situ carcinomas | 3.3 | 2.1 |
| Sleep disorders | 3.3 | 0.2 |
| Chronic eye diseases and blindness | 3.2 | 0.2 |
| Back diseases | 3.0 | 2.2 |
| Diseases of the oesophagus, stomach, and duodenum | 3.0 | 1.8 |
| Other musculoskeletal diseases | 3.0 | 0.8 |
| Inflammatory and other bowel diseases | 3.0 | 0.5 |
| Diabetes | 2.9 | 4.0 |
| Diseases of male genitals/reproductive organs | 2.9 | 3.5 |
| Diseases or arteries and veins | 2.9 | 0.7 |
| Arthrosis | 2.8 | 2.7 |
| Hypertensive diseases | 2.7 | 21.9 |
| Chronic ear diseases and deafness | 2.7 | 0.1 |
| Endocrine diseases | 2.6 | 0.9 |
| Diseases of female genitals/reproductive organs | 2.6 | 0.1 |
| Obesity and other metabolic diseases | 2.4 | 0.7 |
| Chronic skin diseases | 2.4 | 0.6 |
| Chronic diseases of the upper respiratory tract | 2.2 | 0.2 |
